# Preparing for the next pandemic: Simulation-based deep reinforcement learning to discover and test multimodal control of systemic inflammation using repurposed immunomodulatory agents

**DOI:** 10.3389/fimmu.2022.995395

**Published:** 2022-11-21

**Authors:** Chase Cockrell, Dale Larie, Gary An

**Affiliations:** Department of Surgery, University of Vermont Larner College of Medicine, Burlington, VT, United States

**Keywords:** drug repurposing, machine learning and AI, sepsis, multiscale modeling and simulation, agent - based modeling, cytokine storm, COVID - 19, deep reinforcement learning

## Abstract

**Background:**

Preparation to address the critical gap in a future pandemic between non-pharmacological measures and the deployment of new drugs/vaccines requires addressing two factors: 1) finding virus/pathogen-agnostic pathophysiological targets to mitigate disease severity and 2) finding a more rational approach to repurposing existing drugs. It is increasingly recognized that acute viral disease severity is heavily driven by the immune response to the infection (“cytokine storm” or “cytokine release syndrome”). There exist numerous clinically available biologics that suppress various pro-inflammatory cytokines/mediators, but it is extremely difficult to identify clinically effective treatment regimens with these agents. We propose that this is a complex control problem that resists standard methods of developing treatment regimens and accomplishing this goal requires the application of simulation-based, model-free deep reinforcement learning (DRL) in a fashion akin to training successful game-playing artificial intelligences (AIs). This proof-of-concept study determines if simulated sepsis (e.g. infection-driven cytokine storm) can be controlled in the absence of effective antimicrobial agents by targeting cytokines for which FDA-approved biologics currently exist.

**Methods:**

We use a previously validated agent-based model, the Innate Immune Response Agent-based Model (IIRABM), for control discovery using DRL. DRL training used a Deep Deterministic Policy Gradient (DDPG) approach with a clinically plausible control interval of 6 hours with manipulation of six cytokines for which there are existing drugs: Tumor Necrosis Factor (TNF), Interleukin-1 (IL-1), Interleukin-4 (IL-4), Interleukin-8 (IL-8), Interleukin-12 (IL-12) and Interferon-γ(IFNg).

**Results:**

DRL trained an AI policy that could improve outcomes from a baseline Recovered Rate of 61% to one with a Recovered Rate of 90% over ~21 days simulated time. This DRL policy was then tested on four different parameterizations not seen in training representing a range of host and microbe characteristics, demonstrating a range of improvement in Recovered Rate by +33% to +56%

**Discussion:**

The current proof-of-concept study demonstrates that significant disease severity mitigation can potentially be accomplished with existing anti-mediator drugs, but only through a multi-modal, adaptive treatment policy requiring implementation with an AI. While the actual clinical implementation of this approach is a projection for the future, the current goal of this work is to inspire the development of a research ecosystem that marries what is needed to improve the simulation models with the development of the sensing/assay technologies to collect the data needed to iteratively refine those models.

## 1 Introduction

A striking feature of the COVID-19 pandemic in its early phases was that medical resources, particularly those in critical care units, were overwhelmed. This issue arose primarily because of the inability to affect the underlying biological processes that drove the course of disease; once the disease manifested the only option was supportive care until the disease ran its course. Given the challenges in developing specific antiviral agents and the mandatory time required to bring novel drugs or new vaccines to clinical deployment, preparation for the next pandemic should include the development of measures that can more effectively and efficiently use existing drugs to reduce and mitigate disease severity. Specifically, with respect to COVID-19, there was an early recognition that severe disease was associated with “cytokine storm” (1–6) or “cytokine release syndrome” (7–10), namely that the body’s inflammatory/immune response was producing unintended and detrimental collateral damage in response to the viral infection. There is a suggestion that based on the pathophysiological time courses of acute viral infections (1–10) disease manifestation occurs subsequent to the peak(s) of viremia; it is exactly this host-response driven pathophysiological phase that significantly contributes to utilization of in-hospital and critical care resources. As a result, there was a great deal of interest in repurposing immunomodulatory agents to attempt to mitigate disease severity in COVID (11–13), but to date, with the exception of the use of steroids for severe disease (14), none of these approaches have been unambiguously proven to be effective in the treatment of infection-generated cytokine storm/cytokine release syndrome.

This should not come as a surprise. The phenomenon of collateral tissue damage arising from dysregulated inflammation described as “cytokine storm/cytokine release syndrome” is exactly the process that drives disease severity and multiple organ failure in bacterial sepsis, for which no immunomodulatory interventions have been shown to be reliably effective (15). In fact, the current set of immunotherapies for chronic inflammatory diseases, exactly those proposed for repurposed use in COVID, were themselves repurposed from agents that initially failed in sepsis trials. We have previously reported on the challenges present in attempting to control sepsis using anti-cytokine/anti-mediator therapies, primarily stemming from the failures to recognize the dynamic complexity of the mechanistic processes ostensibly being targeted (16) and that in order to be effective the treatment of sepsis should be considered a complex control problem (17). In previous work we have shown that sepsis is potentially controllable by discovering multi-modal control strategies using different types of machine learning (ML) methods trained on a complex agent-based model of acute systemic inflammation (the Innate Immune Response Agent-based Model, or IIRABM (18)) (19–21). Specifically, the latter projects described in Refs (20, 21) utilized the method, Deep Reinforcement Learning (DRL), employed by ML/Artificial Intelligence (AI) systems to successfully play and win a series of games against human experts (22–24). We term this approach *simulation-based DRL*, and in prior work applied to method where we treated the attempt to control sepsis as a “game” to be played using the IIRABM, where potential cytokine interventions represented the “moves” implemented by the AI agent (20, 21).

The recognized heterogeneity seen in clinical cytokine time series data for infection-induced cytokine storm/cytokine release syndrome [for example, as seen in the data from COVID-19 patients (25–30) or those with influenza (31–35)] suggests that individual patients have quite different cytokine profiles at different times, different profiles that may represent different immunological states that may call for different sets of modulators. Differentiation between immunological states is particularly difficult if one is trying to identify that state for an individual patient: for instance, in all the papers regarding time series cytokines in COVID (25–30), while there may be statistical discrimination across the mean population cytokine trajectories there is considerable overlap between individual profiles/values across the range of disease severity classes. This makes it nearly impossible to profile *an individual patient*, which is what is necessary to select among different treatments to decide which particular one should be given. However, different responses to those modulators between otherwise ostensibly similar immune profiles can provide further discrimination of the functional immune responsiveness of an individual, and this response-information can lead to further refinement of what treatment/modulation is called for. All these factors suggest that an adaptive control approach, which simulation-based DRL produces, is called for. We also note that other modeling approaches that utilize differential equations (36–42) have addressed the issue of heterogeneity using purely deterministic methods. Our particular approach is distinct in that data variance arises from both biological stochasticity as well as differential dynamics due to microbial mechanism and host responsiveness.

In the context of developing capabilities to mitigate the disruption of a future potential pandemic, particularly in its early phases where there should be concurrent efforts at developing vaccines and other specific anti-viral agents, we make the following assertions:

1. Dysregulated and detrimental systemic inflammation is a primary source of disease severity in acute viral illness;
2. There is a critical need to have virus-agnostic disease mitigation therapies in the early phases of a pandemic.
3. There is proven inefficacy of standard approaches to applying immunomodulation in the face of cytokine storm/cytokine release syndrome/sepsis; and
4. There is a need to increase the efficiency and efficacy of repurposing existing immunomodulatory agents to provide virus-agnostic disease mitigation options.

We propose that simulation-based control discovery using DRL can provide useful insights and potentially critical capabilities for designing effective multi-modal and adaptive immunomodulatory therapies for infections for which no effective anti-microbial agents exist, as might be expected for a potential pandemic due to a novel infectious agent. This approach requires the generation of synthetic data upon which the DRL can operate. There exist several mechanism-based computational models of the host response is COVID-19 that could theoretically be re-purposed to represent a hypothetical, novel infectious agent; these models are mostly equation-based differential equation models (36–42), with some cases of agent/individual-based models (43) and hybrid models (44). Our work uses the IIRABM for this purpose, and our hope is that our work will prompt other investigators to utilize the presented methods on their models.

We have previously demonstrated in a proof-of-concept report that such a control policy can be discovered with DRL when manipulating up to 11 different mediators and soluble factors every 6 minutes (21). We now extend that study to evaluate whether DRL can train an artificial neural network (ANN) to discover a treatment policy utilizing existing anti-cytokine drugs to improve the outcomes to simulated infection in the absence of anti-microbial treatment. We wish to emphasize that the current investigation is not a simulation specifically of COVID-19, but rather a generic infection able to generate an inflammatory response.

## 2 Methods

### 2.1 Background and rationale for current investigation

The current work is the most recent investigation in a decades-long research program that uses mechanism-based agent-based modeling to address the challenge of controlling infection-induced pathogenic acute systemic inflammation (i.e. sepsis/cytokine storm/cytokine release syndrome); we believe that relaying the path from the initial investigations to the current one will aid in placing the current project in context and help point to future directions. One of the earliest uses of the IIRABM was to perform *in silico* clinical trials of existing (single agent) and hypothetical (including combination therapy) anti-cytokine interventions (18). This work demonstrated that single and simple combination therapy would not work but did not provide a path forward as to what might work; we could not provide guidance as to what might be successful. Over the subsequent decade, with no cytokine manipulating therapies with success in clinical trials, there was concern that it may not actually be possible to control sepsis with mediator manipulation. Therefore, we considered that a more complex multi-modal and potentially variable mediator manipulation might be necessary and used genetic algorithms (GA) to search across all potential combination therapies possible within the IIRABM to determine whether the system could be controlled at all (19). The key findings of this work were that the system was theoretically controllable when using a complex multimodal policy, but differential responsiveness in terms of cytokine trajectories amongst the *in silico* cohort could cause the policy to fail for a subset of individuals. As such, by accounting for the known heterogeneity of inflammatory/cytokine/mediator trajectories our simulations demonstrated an inherent limitation to GA-based optimization. To address this, we attempted to discover “personalized” therapeutic policies using DRL, which is a strategy that inherently accounts for differential paths to similar configurations of the system and optimizes actions to provide the highest probability of the desired outcome. We recognized that this work was at a ‘proof of concept’ stage, and to demonstrate feasibility we allowed full observation and control in order, again, to determine if satisfactory control was even possible (20, 21). In the current work in this paper, we take a step towards clinical relevance by limiting the observation and action space to existing drugs as well as limiting the timing of system measurements and actions in an interval clinically plausible in a critical care environment (e.g. every 6 hours). This is done recognizing that currently the desired assays do not exist; however, we believe that the conceptual demonstration of what might be possible should these sensing technologies exist may help direct advances in sensing technologies, which are a critical piece of the larger puzzle of developing personalizable/adaptable and generalizable therapeutic strategies.

### 2.2 Description of IIRABM

The simulation model used for DRL ANN training is a previously validated agent-based model of sepsis, the IIRABM (16, 18). We have previously used the IIRABM as a surrogate/proxy system for the investigation of potential control strategies (45) for sepsis, both using genetic algorithms (19) and DRL (20, 21). A detailed description of the IIRABM can be found in Ref (18); here we present a brief overview to provide enough background to describe the control discovery work that is the subject of this paper.

**Figure 1:**
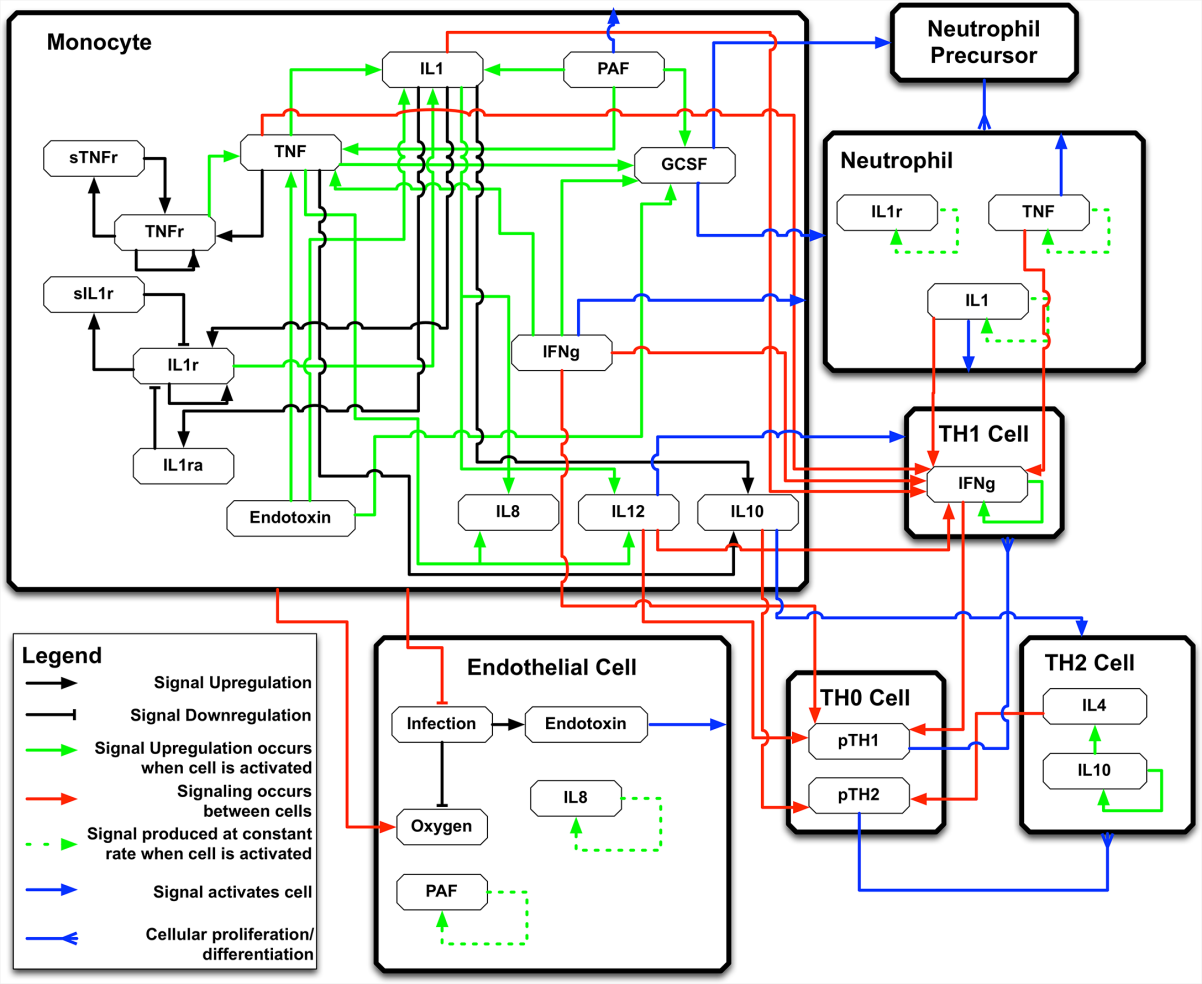
Schematic of cell types, mediator and connections in the Innate Immune Response Agent-based Model (IIRABM). IL1 , Interleukin-1; IL4 , Interleukin-4; IL8 , Interleukin-8; IL10 , Interleukin-10; IL12 , Interleukin-12; TNF , Tumor Necrosis Factor; GCSF , Granulocyte Colony Stimulation Factor; IFNg , Interferon-gamma; TNFr , Tumor necrosis factor receptor; sTNFr , soluble Tumor Necrosis Factor Receptor; IL1r , Interleukin-1 receptor; IL1ra , Interleukin-1 receptor antagonist; pTH1 , pro-Type 1 Helper T-cell state; pTH2 , pro-Type 2 Helper T-Cell state. For full details of the IIRABM see Ref (18). Figure reprinted from Ref (19) under the Creative Commons License.1058478308 (11108).

The IIRABM is a two-dimensional abstract representation of the human endothelial-blood interface with the modeling assumption that the endothelial-blood interface is the initiation site for acute inflammation. The closed nature of the circulatory surface can be represented as a torus, and the two-dimensional surface of the IIRABM therefore represents the sum-total of the capillary beds in the body. The spatial scale of the real-world system is not directly mapped using this scheme. The IIRABM simulates the cellular inflammatory signaling network response to injury/infection and reproduces all the overall clinical trajectories of sepsis (18) and clinically plausible mediator trajectories associated with acute systemic inflammation in response to infection (16, 18, 19). The IIRABM incorporates multiple cell types and their interactions: endothelial cells, macrophages, neutrophils, TH0, TH1, and TH2 cells as well as their associated precursor immune cells. A schematic of the components and interactions in the IIRABM can be seen in **Figure 1**.

The content of the IIRABM is not intended to be a comprehensive list of all the cellular subtypes present in the immune system, but rather represents the minimally sufficient set of cell populations able to represent every necessary function in the innate response to infection. System mortality of the IIRABM is defined when the aggregate endothelial cell damage, represented by the model variable “Oxy-deficit”, exceeds 80% of the total baseline health of the system; this threshold represents the ability of current medical technologies to keep patients alive (i.e., through organ support machines) in conditions that previously would have been lethal.

The IIRABM is a stochastic model, and there are 3 primary sources of “noise” in the IIRABM: 1. The initial spatial distribution of cells across the world grid at initialization (not an unreasonable assumption given that in a particular tissue such variation would be expected to occur) 2. Semi-Brownian movement of cells prior to them encountering a chemotactic gradient (also we believe a not unreasonable assumption) and 3. In the inflection point between competing pathways manifest at the individual cellular level, e.g. the inflammatory state of a macrophage as a weighted ratio of the pro- and anti-inflammatory mediators in the cell’s immediate milieu (also we believe this is not unreasonable). The inclusion of randomness in these three areas is able to generate heterogeneity across a set of simulation runs that mimics the variation seen in clinical populations (16, 18), and allows us to generate virtual populations with defined recovery/mortality rates.

Simulated infections in the IIRABM are initiated using 5 parameters representing the size and nature of the injury/infection as well as a metric of the host’s resilience:

1. Host Resilience: This represents the rate at which a damaged cell recovers its “health”. A higher value means that the host/patient can more rapidly recover from cellular damage/dysfunction.
2. Microbe Invasiveness: This represents the degree to which the microbe can infect surrounding cells. A higher value means that the microbe can more readily infect surrounding cells.
3. Toxigenesis: This represents the degree to which a microbe can damage and eventually kill an infected cell. A higher value means that the microbe can more rapidly kill an infected cell.
4. Environmental Toxicity: This represents the degree of environmental contamination that can lead to nosocomial/hospital acquired infection. This function was not included in the current set of simulation experiments.
5. Initial Infection Size: This represents the amount of the initial inoculum of the microbe.

These 5 parameters represent factors that clearly affect the lethality of a potential infection, but are not functionally extractable or measurable in any reliable fashion. Previous work (16) identified the boundary conditions for these parameters in terms of generating clinically realistic behavior, but for any particular individual these parameters are essentially unquantifiable except in terms of the mortality rate generated within a simulated population. We note that for the current investigations we set the Environmental Toxicity parameter to 0 in order to not confound the simulation experiments by including secondary bacterial infections. Therefore, parameterizations of the IIRABM for these simulation experiments used the parameters (host resilience, microbial invasiveness, microbial toxicity, initial infection size). To set up the DRL training environment, we selected a parameter set that would produce a Recovered Rate of approximately 60% (= 40% mortality), a disease severity roughly approximating that of severe sepsis or viral induced cytokine storm/cytokine release syndrome.

### 2.3 Deep reinforcement learning

Deep Deterministic Policy Gradient (DDPG) (46) was used to discover a control algorithm that is able to heal *in silico* patients by either augmenting or diminishing the concentration of cytokine signaling molecules in the simulation. DDPG is a powerful reinforcement learning (RL) algorithm able to use off-policy data and the Bellman equation (Equation 1) to learn a value function, or Q-function, to determine the most valuable action to take given any state of the simulation.


(1) $$Q^{*}(s,a)=\underset{s'∼P}{E}[r(s,a)+\gamma \underset{a'}{\max}Q^{*}(s',a')]$$

Equation 1: The Bellman Equation.

Value *Q* is a function of the current state and action *(s, a)*, and is equal to the reward *r* from the current state and chosen action *(s, a)* summed with the discounted value of the next state (discount factor = γ) and action *(s’, a’)* where the next state is sampled from a probability distribution *(s’ ~ P).*

The Q-function is discovered through trial and error and allowing an RL agent to optimize the Q-function based on observed rewards from chosen actions. DDPG can be thought of as an extension of the Q-learning algorithm (47), where it is able to choose from a continuous action space. In Q-learning, the next action is chosen from a set of discrete actions that can be taken based on the output of the Q-function. The best action to take from the current state is identified by finding which action will return the highest value from the Q-function. Q-learning is an off-policy algorithm, which means that in the training phase, the RL agent is sometimes able to choose actions that are not the ones chosen by the Q-function. This allows the agent to explore and potentially discover actions that can lead to a greater reward than continuing from an already discovered policy. Q-learning has proven to be very powerful at solving control problems in discrete space and has proven on benchmark RL problems that the discovered control algorithm can be very robust (46).

DDPG extends Q-learning to a continuous action space. It is too computationally expensive to exhaustively search the action space for the optimal action during the learning phase since the action space is continuous. Because of this, DDPG uses an “actor” neural network to choose an action based on the current state. The chosen action is used for the simulation, and a new state and reward is returned by the RL environment. The reward then updates the Q-function to more closely approximate the true value function for the environment, and the updated Q-function is used to perform gradient descent on the actor network to improve its decision making in the future. Because updates to the actor network are made based on an approximation, DDPG is sometimes susceptible to variations in starting conditions, and is sometimes unstable as it learns. Because of this, learning rates for the actor network and the Q-function approximation are usually slow. Additionally, to help stability, DDPG uses what is called an “Experience Replay Buffer” to sample a batch of states and actions the agent has taken in the past, instead of relying only on the current state and action for network updates.

#### 2.3.1 Training environment

The goal of this work is to determine if an effective immunomodulatory strategy utilizing a set of existing anti-cytokine drugs could balance the need for an effective immune response to contain an infection in the absence of anti-microbials while preventing system death due to cytokine storm. We wanted to mimic a clinically relevant population and therefore chose parameter values and initial conditions to provide an overall mortality of ~40%. Using our previously identified method of finding relevant parameter sets within bioplausible parameter space (16) we chose the following parameters and initial infection level in order to identify the baseline conditions of the IIRABM that would subsequently be used for DRL:

• Host Resilience [oxyheal] = 0.08: This represents the rate at which the baseline endothelial cells recover their oxy level, back to a baseline of 100.
• Invasiveness [infectSpread] = 2: This represents the number of adjacent grid spaces the infection spreads to after it has reaches the carrying capacity on an individual grid.
• Environmental Toxicity [numRecurInj] = 0: This represents the number of grid spaces are randomly reinfected every 24 hours, reflecting environmental contamination. In these set of simulation experiments this function was not included.
• Toxigenesis [numInfectRep] = 2: This represents the amount of damage produced by a microbe on the grid space it occupies.
• Initial Infection Amount [inj_number] = 20: This represents the radius in number of grid spaces of a circular inoculation of the infection

With these parameters the IIRABM had a Recovered Rate of 61% (= 39% Mortality) at the end of simulated 21 days. We note that the process of DRL requires choosing a single parameter set for training and internal testing; we then evaluated the generalizability of the learned treatment policy by testing it on a series of additional parameterizations.

##### 2.3.1.1 Initial and termination conditions

A training episode begins 12 hours after the application of the initial infection; this is to reflect the minimal necessary incubation time between exposure and initiation of any treatment. The episode ends when either the simulated patient completely heals, dies, or if 500 time steps (= 21 simulated days) have passed without meeting stopping conditions.

##### 2.3.1.2 Observation space

The IIRABM states exists over a discrete, 2-dimensional 101 x 101 grid. The IIRABM includes 9 cytokines, 2 soluble cytokine receptors (essentially inhibitors of their respective cytokines), and population levels of 5 different cell types. The IIRABM also reports the total amount of infection in the system and the total amount of damage present in the system (as reflected by the variable “Oxy-deficit”; but for purposes of this paper this term will be called “Total System Damage” for enhanced clarity). Since the IIRABM utilizes an abstract spatial representation, the individual discrete grid cells are not directly translatable to any potential spatial measurement. The aggregated system levels are considered equivalent to values potentially sampled in the blood, and therefore represent the accessible information for any potential sensor or lab assay. As this work attempts to approximate what might eventually be available clinically, we assume that any circulating cytokine/soluble receptor can be measured and returned every 6 hours: this gives the system state as reflected in 11-dimensions (e.g. 9 cytokines + 2 soluble receptors represented in the IIRABM, hereafter termed “mediators”). Alternatively, since in the clinically setting there is a distinction between infectious particles in the tissue and those that spill over into the blood, we do not consider the total system infection a clinically feasible observable and this value is not included in the observation space for the DRL. Similarly, since the total amount of damage in the system is not actually a quantifiable or observable metric in the clinical patient, this value is not included in the observations used to train the DRL; this is in contrast to our prior use of DRL trained on the IIRABM (20). As such, the current DRL agent is being trained on partially-observable states of the IIRABM.

##### 2.3.1.3 Action space

In prior work we have examined whether acute systemic infection due to an infectious agent for which no effective antimicrobials exist is controllable at all through immunomodulation (21). That work, intended to determine if such control was feasible at all, used the most extreme observation and control space possible given the resolution of the IIRABM: every mediator in the IIRABM could be manipulated up or down every 6 minutes. The findings from this paper (21) suggested that it was possible to reduce mortality from 85% to 10.4%. The current work represents a step towards greater clinical plausibility, and restricts the action space both in terms of interval and number of mediators manipulated. Therefore, the current actions taken by the DRL agent can either be augmentation or inhibition of six mediators present in the IIRABM for which there are existing FDA-approved pharmacological agents: Tumor Necrosis Factor-alpha (TNF), Interleukin-1 (IL-1), Interleukin-2 (IL-2), Interleukin-4 (IL-4), Interleukin-8 (IL-8), Interleukin-12 (IL-12) and Interferon-gamma (IFNg). As this study is a proof-of-concept for potential clinical plausibility, we choose a hypothetical yet clinically plausible time frame in which a potential blood mediatory assay would be run and used to inform the administration of a drug/set of drugs of 6 hours; therefore, for the DRL action space interval, any or all of these mediators could be manipulated every 6 hours. For this proof-of-concept study and given our experience that controlling infection-induced acute systemic inflammation is a complex control problem this restriction of the observation and control space represents a step towards a more potentially clinically-relevant simulation experiment. As a simplifying approximation of clinical pharmacological effect the duration of the effect of each intervention was simulated to last for 6 hours. An augmentation action takes the form of the addition of a continuous value from 1 to 10 to the value of a particular mediator. Inhibition takes the form of the multiplication of the existing mediator value by 0.001 to 1; this approach is done to avoid negative (or exploding, in the case of pathway augmentation) values and is consistent with the dynamics of mediator inhibition. These are reflected in the code thusly: if action_mag > 0, action = (action_mag) +1 ⇒ add mediator between 1 and 10; if action_mag<0, action = action_mag + 1.001 ⇒ multiply mediator between 0.001 and 1.

The ability to manipulate any combination of mediators present is meant to simulate the potential use of combinations of interventions, which our prior work has suggested is necessary to effectively control sepsis (19–21); the DRL approach is intended to assist in addressing the exponential/combinatorial issues associated with multi-drug therapy and the additional challenge needing to modify a particular treatment application to account for the temporal heterogeneity among individuals with regards to their disease trajectories.

##### 2.3.1.4 Reward function

The current DRL strategy includes two types of reward functions. The first are *terminal rewards*: these are evaluated at the end of an episode and are analogous to either winning or losing the game. The current work has a positive terminal reward if the system heals: *r*=0.999^step^*1000, whereas the negative terminal rewards if the system dies is: *r*=0.999^step^-1000. The incorporation of the step at which the terminating condition is met is intended to reward quicker healing, penalize faster death, and not penalize prolongation of life (albeit in a diseased state). The current work also includes *intermediate or step-wise rewards*; these are reinforcing conditions to aid in learning during the course of the episode run. The intermediate reward function is:


(2) $$OD_{t-1}-OD_{t}-\sum_{i}\left|a_{i}\right|$$

where *OD_t_* indicates the total system damage at time *t*, and *α_i_* is the value for the action taken on mediator *i*. The intermediate reward calculation rewards systems that reduce their damage per time step and are able to do so with a minimal amount of intervention. The latter goal is consistent with the concept of minimizing necessary interventions and avoiding potential side effects that may not be reflected in the resolution of the simulation. Note that while we do not include the total system damage as an observable that can be used to determine actions, we believe it is valid to include it in terms of the reward function since this is a property of the simulation-based training and not intended to reflect a clinically-accessible metric.

##### 2.3.1.5 Training procedure

For training a batch of 20 DDPG agents were created using the stable_baselines3 package and trained simultaneously using MPI on the IIRABM environment. The environment is set up such that the agent selects an action, and that action is held for 6 hours, then the agent is free to select a new action based on its observation. The simulation ends when oxygen deficit (a measure of total system damage in the IIRABM) either reaches a value below 100 (indicating a fully healed run) or above 8160 (indicating damage so great that the run is considered dead). If the simulation does not reach a terminal conclusion by 5000 simulation steps (~21 days of simulated time), the episode is cut off and a new one is started. The reward at each step is the change in oxygen deficit compared to the previous step, with a decreasing value returning a positive reward. A terminal reward of 1000 is given for a run ending with a successful heal, and a penalty of -1000 is given for a run ending with a death. Each agent was allowed to take 432,000 actions during the learning period, which equates to at least 6000 episodes, with that amount increasing slightly depending on how many episodes end before the time limit of 4200 simulation steps.

The code for the DRL environment (which includes the IIRABM and the DRL training code) can be found at https://github.com/An-Cockrell/DRL_Control

## 3 Results

There were three possible outcomes from the simulations under control conditions: 1) Complete healing, where the Total System Damage goes to 0; 2) System Death, where the Total System Damage reaches 80% of baseline health (arbitrarily set and consistently used since initial paper on the IIRABM in 2004 (18)); and 3) “Time Out” where the system achieves neither condition #1 or #2. We note that the Time Out condition is not present in the baseline uncontrolled case, where simulated individuals either Recovered (61%) or Died (39%) by the end of the simulation run (5,000 steps = ~21 days). The Time Out result arises from the application of the control policy to the simulation such that the control provides “life support” that prolongs the duration of a system run that would likely otherwise “die”. We identified that below a threshold of Total System Damage of 600 the system would invariably heal, and therefore aggregated our outcomes into two groups; “Recovered” which includes completely healed and those with Total System Damage< 600 at the end of 21 days simulated time, and “Non-recovered” for those simulations that met Death criterial (Total System Damage > 80%) or Total System Damage > 600 at the end of 21 days. Incidentally, this baseline mortality rate is approximately that of COVID-19 in the pre-pharmacological treatment era.

**Figure 2:**
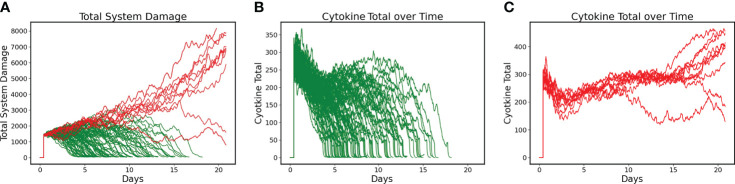
Differences between Recovered and Non-recovered Groups in terms of Total System Damage and Levels of Total Cytokines/Mediators present in the system. Simulations run for 21 days. **(A)** Total System Damage Trajectories with DRL control policy. N = 100, Recovered (Green) = 90, Non-Recovered (Red) = 10. **(B)** Trajectories of Total Cytokines/Mediators under control policy for the Recovered Group (N = 90). **(C)** Trajectories of Total Cytokines/Mediators under control policy for the Non-recovered Group (N = 10). Comparing Panels B and C it is evident that worse outcome was associated with sustained activation of the system’s pathways, lending intuitive support for treatment policies that focus on mediator inhibition.

DRL training proceeded for 6000 episodes and converged to a policy that had a Post-Control Recovered Rate = 90% with a Non-recovered Rate of 10% (N=100). These results were a significant improvement over the uncontrolled base condition, which had a Recovered Rate = 61% (Non-recovered rate of 39%). **Figure 2A** shows the Total System Damage Trajectories between simulated patients that Recover (Green) and those that do not (Red). Panels 2B and 2C show the total level of cytokines/mediators present in the system in both the Recovered (**Figure 2B**) and Non-Recovered (**Figure 2C**) groups. It is evident that worse outcome was associated with sustained levels of pathway activation, which corresponds to the hypothesis that hyperactivation of inflammation is a significant driver of disease severity.

**Figure 3:**
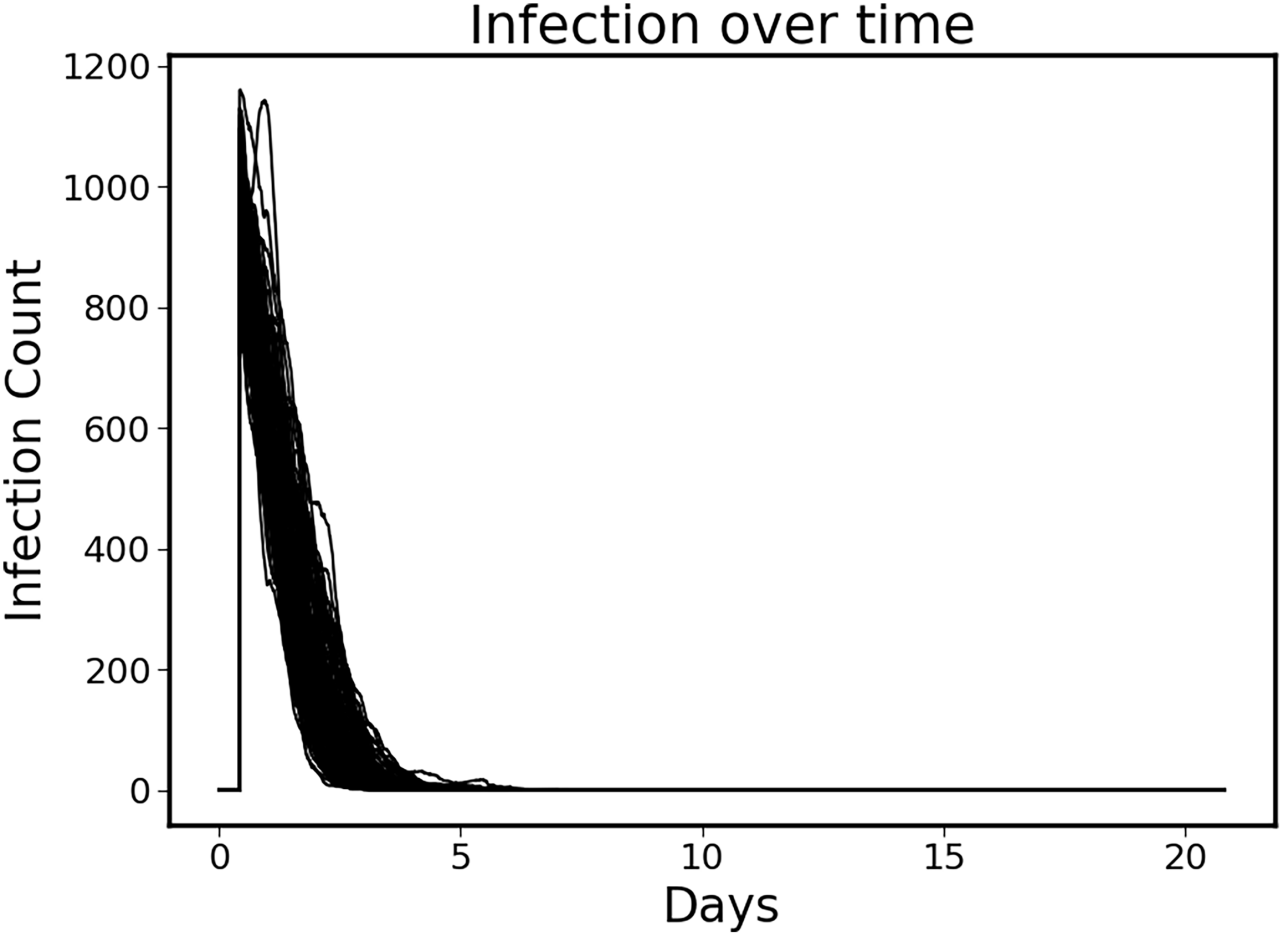
Clearance of initial infection via controlled immune functions. In all circumstances the initial infection was controlled by modulating the 6 targeted mediators, with infection essentially eradicated by ~Day 6.

The control policy was also able to completely eradicate the initial infection without the aid of antimicrobials by augmenting the immune clearance capability (**Figure 3**). Note that the infection is essentially eradicated by ~ 6 days.

**Figure 4:**
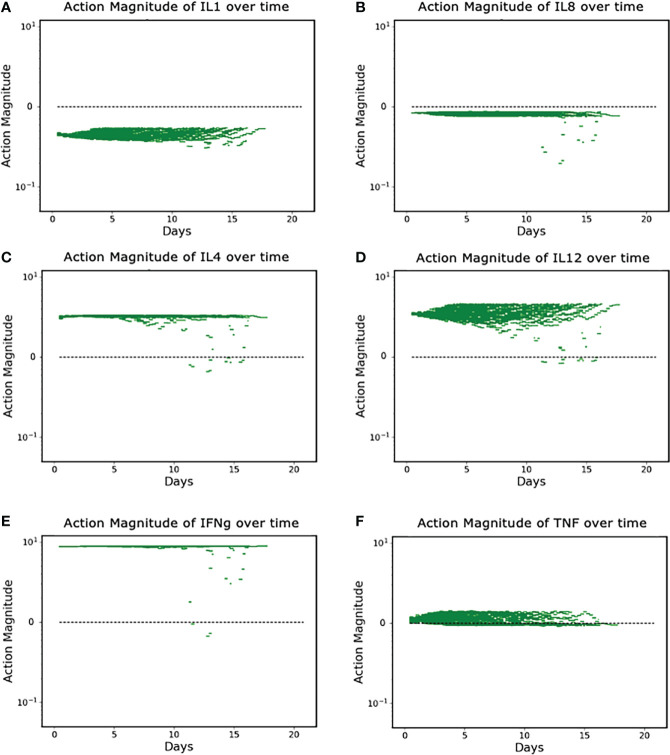
**(A–F)**. Control Actions taken in Recovered Group (N = 90). **(A, B)** show control actions that always inhibit IL1 and IL8. Panels **(C–E)** show control actions that primarily augment IL4, IL12 and IFNg; **(F)** shows control actions that can vary from augmentation to inhibition for TNF. Note Y-axis is log scale.

The discovered policy manipulated each of the six targeted mediators in some fashion (either augmentation or inhibition) every six hours in a variable fashion. Successful control outcomes in the Recovered group are seen in **Figures 4A-F**. These actions can be broadly divided into three classes: constant inhibition, constant augmentation, and balance/optimization.

Panels A and B show the cytokines whose protein synthesis pathways are constantly inhibited, IL1 and IL8; Panels C, D, and E show the (near) constant augmentation of protein synthesis pathways for IL4, IL12 and IFNg; Panel F demonstrates the algorithm “balancing” the appropriate level of TNF in order to optimize the outcome. We note that the (near) constant augmentation actions contain very few and very brief periods of inhibition. Given that excess inflammation can lead to additional tissue damage or runaway inflammation, we speculate that these rare and brief periods of inhibition are the algorithm attempting to “pump the brakes” on the inflammatory process and maintain sufficient inflammation to heal infection and prevent further nosocomial infections while preventing the inflammation from progressing to a state in which it is uncontrollable. TNF is distinct in that the actions contain both augmentation and inhibition throughout the duration of the controlled simulation. This is a more extreme example of the logic outlined above – additional pro-inflammatory stimulus (i.e., TNF) is needed to successfully heal the system, but too much will lead to a negative outcome.

**Figure 5:**
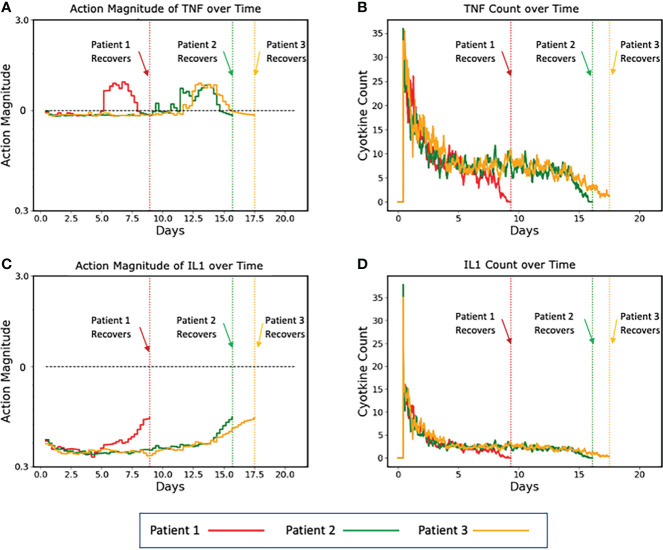
Plots of three representative individual controlled simulated patients, all of which recover, showing control actions **(A, C)** and targeted mediators being controlled **(B, D)**. **(A, B)** show the control actions on TNF and the resulting trajectories of TNF, respectively. Panels C and D show the control actions on IL1 and the resulting trajectories of IL1. The different dynamics between the three patients can be seen in the variation between their individual control actions and targeted mediators. Note that each of these simulated patients recovers at a different time point (vertical dashed lines), at which time their respective simulations terminate. Also note that Y-axis is plotted using absolute values (not log scale as seen in **Figure 4**).

To aid in visualizing how the implementation of a control policy addresses variability/heterogeneity in cytokine dynamics seen between individuals, the following graphs show the specific control trajectories for three specific simulated individuals who were successfully treated (**Figure 5**).

In this figure, the balancing behavior of the TNF control is especially apparent. We note that the action magnitude lies just below the neutral line for the majority of the duration of the simulation, interrupted by brief periods of augmentation near the terminus of the simulation, which is needed to completely resolve the initial insult. Similar behavior is shown for the IL1 control, however instead of augmentation near the end of the simulation, it lessens the magnitude of inhibition. While these alterations of actions were likely not necessary for the system to heal in general, they did optimize the outcome – they healed the system quicker than if no action were taken. Additionally, because these augmentations (or decreased inhibitions) occurred when the system configuration was in a less inflammatory state (i.e., the serum cytokine concentration of pro-inflammatory molecules was significantly less after some healing had occurred), the danger of augmentation leading to runaway inflammation was ameliorated.

Lastly, in order to test whether the learned control policy was applicable to other IIRABM parameterizations we tested the efficacy of the control policy for 4 additional conditions:

• Test 1: Host Resilience = 0.1: Invasiveness = 1:Toxigenesis = 3: Initial Injury = 20. Notation Label (0.1, 1, 3, 20). Test 1 Parameters generated a Baseline Recovered Rate = 25%. This parameterization represents a group with higher health resilience (corresponding to better baseline health status), but exposed to a microbe that more rapidly kills infected cells.
• Test 2: Host Resilience = 0.12: Invasiveness = 1:Toxigenesis = 1: Initial Injury = 32. Notation Label (0.12, 1, 1, 32). Test 2 Parameters generated a Baseline Recovered Rate = 16%. This parameterization represents a group with even higher baseline health, but is exposed to a much larger initial inoculum (demonstrating dose-dependent disease severity).
• Test 3: Host Resilience = 0.08: Invasiveness = 2:Toxigenesis = 1: Initial Injury = 23. Notation Label (0.08, 2, 1, 23). Test 3 Parameters generated a Baseline Recovered Rate = 19%. This parameterization represents a group with the same baseline health status as the training set, but was exposed to a microbe that more readily infects surrounding cells.
• Test 4: Host Resilience = 0.12: Invasiveness = 2:Toxigenesis = 1: Initial Injury = 28. Notation Label (0.12, 2, 1, 28). Test 4 Parameters generated a Baseline Recovered Rate = 37%. This parameterization represents a group with higher baseline health, but was exposed to a microbe that more readily infects surrounding cells.

The results of applying the previously trained DRL-policy is shown in **Table 1**. We found that the learned policy is broadly generalizable across a suite of microbial insults with a range of inherent microbial properties (e.g., degree of invasiveness, toxicity effects), with improvements in Recovered Rate ranging from +33% to +56%.

**Table 1:** Therapeutic model generalizability.

Parameterization	Uncontrolled Recovery Rate	Controlled Recovery Rate	Improvement
Test 1: (0.1,1,3,20)	25%	81%	56%
Test 2: (0.12,1,1,32)	16%	56%	40%
Test 3: (0.08,2,1,23)	19%	52%	33%
Test 4: (0.12,2,1,28)	37%	83%	46%

The baseline and controlled mortality rates (MR) for IIRABM parameterizations upon which the DRL algorithm was *not* trained are presented here. Parameterizations are defined as (host resilience, microbial invasiveness, microbial toxigenesis and initial injury size).

## 4 Discussion

Future pandemics are an inevitability, and while many preparations for future pandemics focus on somehow enhancing novel drug/anti-viral/vaccine development (important as they are), there are certain points that are worth noting:

1. Pathogen-agnostic disease mitigation is a critical capability in terms of readiness for future viral pandemics. While there is a certain appeal to developing viral-species specific interventions, such as anti-viral agents and vaccines, these agents have a mandatory lag-time in terms of their development; despite the impressive and unprecedented success and rapidity of COVID-19 vaccine development, it is difficult to imagine how such modalities could be made available is less than a year. Alternatively, there is a highly conserved mechanism of disease pathogenesis arising from the host inflammatory response, a shared feature of many viral infections (1–6). Developing effective strategies to control this process, while maintaining host capability to eradicate the infection, would provide a crucial capability in the early phases of any future pandemic.
2. However, the need to balance effective inflammatory/immune antimicrobial responses while mitigating the detrimental effects excessive inflammation is a highly complex task. Sepsis has been known to involve disordered and “excessive” inflammation for half a century (48). However, attempts to modulate the inflammatory response in the face of acute infection ever since have failed to effectively translate into the clinical arena (16). COVID-19 resurrected this interest (49), with what should have been expected undecisive results. The general failure of immunomodulation in the face of acute infection suggests that future approaches should consider this problem as complex control problem, and apply methods appropriate to solving complex control problems (17).
3. Drug repurposing is not as simple as extrapolating the putative mechanism of a drug and assuming that such a mechanism would be efficacious in a completely different context. The urgency of COVID-19 prompted the initiation of multiple potential therapies and trials based on bioplausibility; but it should be noted that every failed clinical trial presupposes that same bioplausibility. The same Translational Dilemma present in the development of new therapeutics (50) is also in play with the drug repurposing task, and requires the same readjustment of how to accomplish that task. Notably, the nature of the Translational Dilemma, i.e., the need to dynamically mechanistically-evaluate putative mechanistic bioplausibility, means that correlative approaches that utilize AI/traditional computational approaches do not provide a scientifically sound path that addresses the fundamental step in the drug evaluation process because they rely on correlative methods and the extrapolation of mechanistic-effect that has been demonstrated to be ineffective (51–53).

We have previously proposed that the integration of advanced forms of ML (specifically DRL) and high-fidelity mechanism-based simulations provides a scientifically sound path forward (17, 20, 54). As noted in the section “Background and Rationale for the Current Investigation” the trajectory of our preceding work started from an attempt to address the lack of clinical success in effectively controlling sepsis by attempting to answer the question: “Is sepsis controllable?” Having demonstrated through a series of proof-of-concept methodological studies that controlling sepsis is conceptually possible, we now move in the direction of using those methods to identify more clinically translatable findings. This current study takes the first steps in that direction by limiting the control space to clinically available drugs within a clinically plausible interval, and suggests that this is also conceptually feasible. However, we recognize that even the intervention policy proposed herein may not be completely necessary, and therefore future studies will involve modifying and evolving our methods to identify minimally-sufficient control policies, such as fewer combinations of drugs and less frequent assay-treatment intervals, that would be more translationally tractable. The intent of this approach is to provide a systematic framework that can aid in directing the development of future sensor and assay technology by identifying boundary conditions for their capabilities and identifying new classes of drugs/targets while utilizing those compounds that already exist.

Another potential benefit of the use of simulation-based DRL is that the existence of the mechanism/knowledge-based simulation model provides a degree of interpretability for an artificial intelligence. The ability to examine the control policy in reference to the mechanistic target of the control and the affected behavior of the simulation model (see **Figures 4**, **5**) provide a means of positing why a particular strategy works and can potentially help overcome concerns regarding the “black box” nature of most modern AI systems.

A clear and critical challenge moving forward is developing more detailed and trustworthy simulation models that can be used for training AI-controllers that can be clinically deployed. We recognize that there are multiple directions in which the IIRABM can be enhanced, and we are actively exploring refinements such as adding in additional T-cell subtypes (including TH17 cells), other aspects of the adaptive immune response, representing local physical processes such as the development of tissue edema, and integrating with physiological models able to generate systemic phenomena such as cardiovascular metrics (such as blood pressure and cardiac output) and pulmonary measurements (such as oxygenation/gas exchange). Achieving the goal of developing more trustworthy simulation models includes technical challenges that include not only being able to represent the biology in sufficient detail, but developing methods for calibrating and parameterizing such models that take into account the inherent incompleteness of biological knowledge and the considerable heterogeneity seen in biological behavior (55, 56). Inherent to this process is also identifying appropriately complex *in vivo* models in which conceptually effective control strategies can be tested in a real-world system; we believe this capability would require finding collaborators with expertise with large-animal “ICU” models able to implement the cycle of sense/control/sense suggested by the DRL. Our hope is that the dissemination of this work will aid in this process.

The need to deal with a perpetual and inevitable incompleteness of mechanistic knowledge is a key point that also needs to be recognized and dealt with: it cannot be that we must wait to know “everything” about how the biology works before we can hope to engineer interventions. Rather, we must recognize the need to develop paths forward that can provide some clinical utility while building in the capabilities to perpetually and iteratively improve and refine our simulation models. An example of this structure can be seen in the evolution of weather modeling and prediction: when the importance of being able to predict hurricane behavior was identified in the 1950s it was well-recognized that the existing mathematical models were insufficient for the task. But rather than saying this extremely difficult task was not worth pursuing, a model-driven data collection ecosystem was developed with the explicit goal of both providing some then-present-day benefit (limited though that may have been), as well as, more importantly, identifying and collecting the *type of data needed to improve the models*. In this case, the modeling proposed was not limited by what sort of data could be collected; rather, the types/scale/complexity of the models needed to solve the problem were specified, and data collection strategies and capabilities were developed to allow the construction of the necessary models. This is the inflection point that biomedical community faces today in terms of fully leveraging the potential of mechanism-based, algorithmic simulation models (54, 57). We hope that the proof-of-concept demonstration presented in this manuscript will provide additional stimulus at the potential for the role of mechanistic algorithmic simulation models and how those models can be integrated with cutting edge ML and AI methods. We hope that this work will prompt additional investigations to improve and advance this methodology, and, critically, help drive the corresponding developments in real-time mediator/cytokine sensing and administration such that we will be better prepared to face the next inevitable pandemic.

## Data availability statement

The raw data supporting the conclusions of this article will be made available by the authors, without undue reservation.

## Author contributions

CC conceived of the paper, helped design and perform simulation experiments, helped with the analysis and contributed to the preparation of the manuscript. DL performed the simulation experiments and analysis and contributed to the preparation of the manuscript, GA helped conceive of the paper, helped design the simulation experiments, helped with the analysis and contributed to the preparation of the manuscript. All authors contributed to the article and approved the submitted version.

## Funding

This work was supported in part by the (NIH) Award UO1EB025825. This research is sponsored by the Defense Advanced Research Projects Agency (DARPA) through Cooperative Agreement D20AC00002 awarded by the U.S. Department of the Interior (DOI), Interior Business Center. The content of the information does not necessarily reflect the position or the policy of the Government, and no official endorsement should be inferred.

## Conflict of interest

The authors declare that the research was conducted in the absence of any commercial or financial relationships that could be construed as a potential conflict of interest.

## Publisher’s note

All claims expressed in this article are solely those of the authors and do not necessarily represent those of their affiliated organizations, or those of the publisher, the editors and the reviewers. Any product that may be evaluated in this article, or claim that may be made by its manufacturer, is not guaranteed or endorsed by the publisher.
